# Lumps and Bumps of the Gingiva: A Pathological Miscellany

**DOI:** 10.1007/s12105-019-01000-w

**Published:** 2019-01-29

**Authors:** Daniel J. Brierley, Hannah Crane, Keith D. Hunter

**Affiliations:** 1 Academic Unit of Oral and Maxillofacial Medicine and Pathology, School of Clinical Dentistry, University of Sheffield, Claremont Crescent, Sheffield, S10 2TA UK; 2 Department of Oral Pathology and Biology, University of Pretoria, Pretoria, South Africa

**Keywords:** Epulis, Gingiva, Fibrous hyperplasia, Giant cell granuloma, Pyogenic granuloma, Ligneous gingivitis, Granulomatosis with polyangiitis, Benign tumor, Malignant tumor

## Abstract

Lesions of the gingivae are amongst the commonest lesions seen in patients and the vast majority are reactive hyperplasias, related to a number of chronic irritant stimuli. However, there are a number of entities that have a predilection for the gingivae, which are much less common in other parts of the oral cavity. The purpose of this paper is to discuss the clinical and histological differential diagnoses when presented with a lump on the gingivae, including the approach to diagnosis and diagnostic pitfalls.

## Introduction

Lesions of the gingiva are very common and provide a significant proportion of the diagnostic workload of any oral pathology practice. The majority of these lesions are reactive (with varied appearance), but other developmental and neoplastic conditions can also present in the gingiva, giving rise to areas of clinical and histological uncertainty in diagnosis. In this review, we aim to address the main entities which may present as “lumps and bumps” in the gingiva. As with all common diagnoses, there is variability in the clinical presentation of such lesions of the gingivae and to some extent, in the histological appearances. As such, it is not possible to describe every possible presentation. However, the descriptions below will cover the most salient features of a range of different pathologies of the gingival tissues.

## A Word on Terminology

As in many areas of histopathology, terminology is variably used and those used in the description of clinical and histological lesions of the gingivae are no different. Some use of terminology is, strictly, inaccurate, but has, for a number of reasons, become well established as common usage. This includes, but is not limited to, the terms ‘polyp’ and ‘epulis’ as histological diagnoses, the use of the term ‘fibroma’ in the context of these lesions, and the common usage in some parts of the world of the term ‘peripheral ossifying fibroma’, which causes confusion in others [1, 2]. Whilst it is not helpful to be dogmatic about which terms should or should not be used, it is important that histopathologists and referring clinicians have a common understanding of the terminology, to ensure effective communication of diagnosis and resulting treatment strategies. Where appropriate, synonyms will be indicated in the text.

## Reactive Lesions

### Fibrous Hyperplasia of the Gingiva

#### Epidemiology

Nodules of inflammatory fibrous hyperplasia (syn. fibroepithelial polyp, fibroma; on gingivae, fibrous epulis) are very common, with fibrous hyperplasia accounting for up to 40% of mucosal pathology in large series [3]. Lesions occur over a wide age range and are more common in females.

#### Clinical Presentation and Differential Diagnosis

**Fig. 1:**
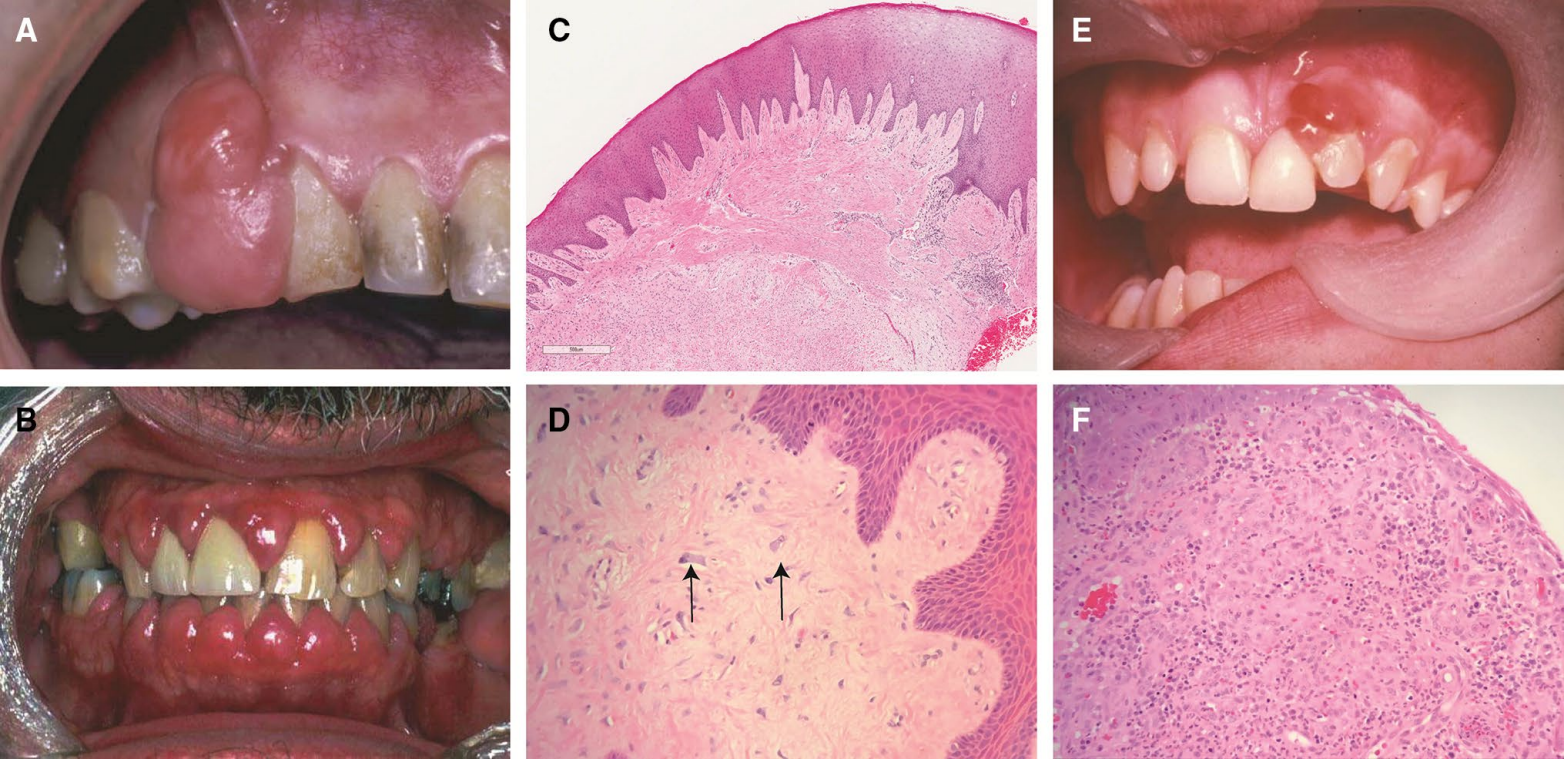
**a** A large fibrous epulis on maxillary gingiva. **b** Widespread fibrous gingival enlargement on a patient on cyclosporine therapy. **c** Histological image of a nodule of fibrous hyperplasia of the gingiva (H&E, Overall magnification × 20). In this case, the collagen varies from superficially hyalinised to more edematous in deeper tissues. **d** Histological image showing large stellate fibroblasts in a giant cell fibroma (H&E, overall magnification × 200). **e** An ulcerated vascular lesion on the maxillary gingiva of a pregnant patient in mid-trimester. **f** The histology of a vascular epulis/pyogenic granuloma shows attenuated or ulcerated epithelium with an underlying endothelial proliferation. This may have a lobular pattern (H&E, overall magnification × 200)

In dentate patients, these lesions most commonly occur on the interdental papilla, but may also include the facial surface of the tooth (Fig. 1a). When large, lesions may extend through the contact point to appear in the papilla on both sides of arch, with a rather “dumb-bell” appearance, although this appearance is more commonly seen in peripheral giant cell granuloma (see below). The lesions are most commonly mucosal colored, but may be focally ulcerated. More extensive lesions occur in patients taking certain medications, for example: phenytoin, nifedipine (and other calcium channel blockers) or cyclosporine [4]. This drug-induced gingival hyperplasia is an exaggerated form of the more focal reactive lesions described above (Fig. 1b). In edentulous/partially dentate patients, similar lesions can occur on the alveolus in relation to the presence of ill-fitting prostheses, often termed denture irritation hyperplasia or denture hyperplasia [5]. These lesions are most common in the mucosa in contact with the periphery of a denture and are usually broad based leaf-like folds of mucosa.

The clinical differential diagnosis most often includes other reactive lesions of the gingiva including pyogenic granuloma and peripheral giant cell granuloma. Considerations in more generalised lesions include hereditary gingival fibromatosis, which may occur as an isolated lesion or as part of a syndrome. A number of genetic lesions, including mutations of the Son-of-Sevenless-1 (SOS1) gene have been associated with the isolated from of this condition [6].

#### Histology and Histological Differential Diagnosis

Histology shows hyperplastic and usually keratinized epithelium overlying nodular fibrous connective tissue (Fig. 1c). The extent of collagenisation and vascularity of the body of the lesion will depend on its maturity and the presence or absence of inflammation. The fibroblast component is bland and in most cases comprises fine spindle shaped cells, with most lesions relatively paucicellular. However, in some cases, the fibroblasts may be large and stellate in morphology, and occasionally multinucleated, albeit cytologically bland (Fig. 1d). These lesions have been termed “giant cell fibroma” and are most common on the gingiva of young adults.

Up to a third of such lesions on the gingiva contain trabeculae of metaplastic bone, particularly those on the maxillary labial gingiva, and which are or have been ulcerated [1, 7]. Such lesions are termed peripheral ossifying fibroma (synonym: mineralizing fibrous epulis). The use of this terminology varies geographically, as does the viewpoint that this represents a separate diagnostic entity. In their survey of reactive gingival lesions, Eversole and Rovin indicated that this lesion most likely represents a variation in response to chronic irritation [8]. Histologically, the mineralizing component consists of trabeculae or drop-like calcifications resembling woven bone or cementum in a background of active cellular stroma. The recurrence rate of such lesions is higher than for other forms of fibrous epulis [7].

### Vascular Epulis

#### Epidemiology

The vascular epulis (syn. pyogenic granuloma) is a reactive vascular lesion, which, in the mouth, is most common on the gingiva. These lesions develop as a result of trauma or recurrent irritation, most commonly in females [9]. The development of these lesions is more common in circumstances where alterations in sex hormones levels are present, for example; in puberty, pregnancy (syn. pregnancy epulis; granuloma gravidarum) and effects of the oral contraceptive pill or hormone replacement therapy [10].

#### Clinical Presentation and Differential Diagnosis

Vascular lesions of the gingiva present as soft bright red swellings that may also have, in areas, a grey/yellow tinge due to the presence of ulceration (Fig. 1e). Hemorrhage is easily provoked on minor trauma. The clinical differential diagnosis includes peripheral giant cell granuloma, as both lesions tend to be vascular in nature. Generalized vascular lesions of the gingiva are unlikely to be vascular epulis/pyogenic granuloma. Consideration should be given to systemic causes of vascular expansion of the gingiva, such as leukemia and granulomatosis with polyangiitis.

#### Histology and Histological Differential Diagnosis

Histology shows a proliferation of endothelial cells, arranged in sheets or as small capillaries, often with a lobular architecture (Fig. 1f). Some lesions contain larger, dilated, thin-walled vascular spaces. The background connective tissue is often loose and oedematous and there may be significant red cell extravasation. The surface epithelium is often ulcerated. In most cases, the diagnosis is straight forward, particularly if a lobular architecture is present. However, some lesions may consist of solid islands of endothelial cells, with a significant mitotic rate. Thus care must be taken not to miss a more sinister lesion on the gingiva, or, conversely, to overcall these features in a benign condition.

### Peripheral Giant Cell Granuloma

#### Epidemiology

The peripheral giant cell granuloma (PGCG) (syn. giant cell epulis) accounts for approximately 10% of epulides [11]. They occur over a wide age range with a lower age peak incidence for males than females, and a female predilection. These lesions can occur in any part of the gingiva in dentate patients or on the alveolar ridge in edentulous patients, but most occur anterior to the molar region and are slightly more common in the mandible [12].

#### Clinical Presentation and Differential Diagnosis

**Fig. 2:**
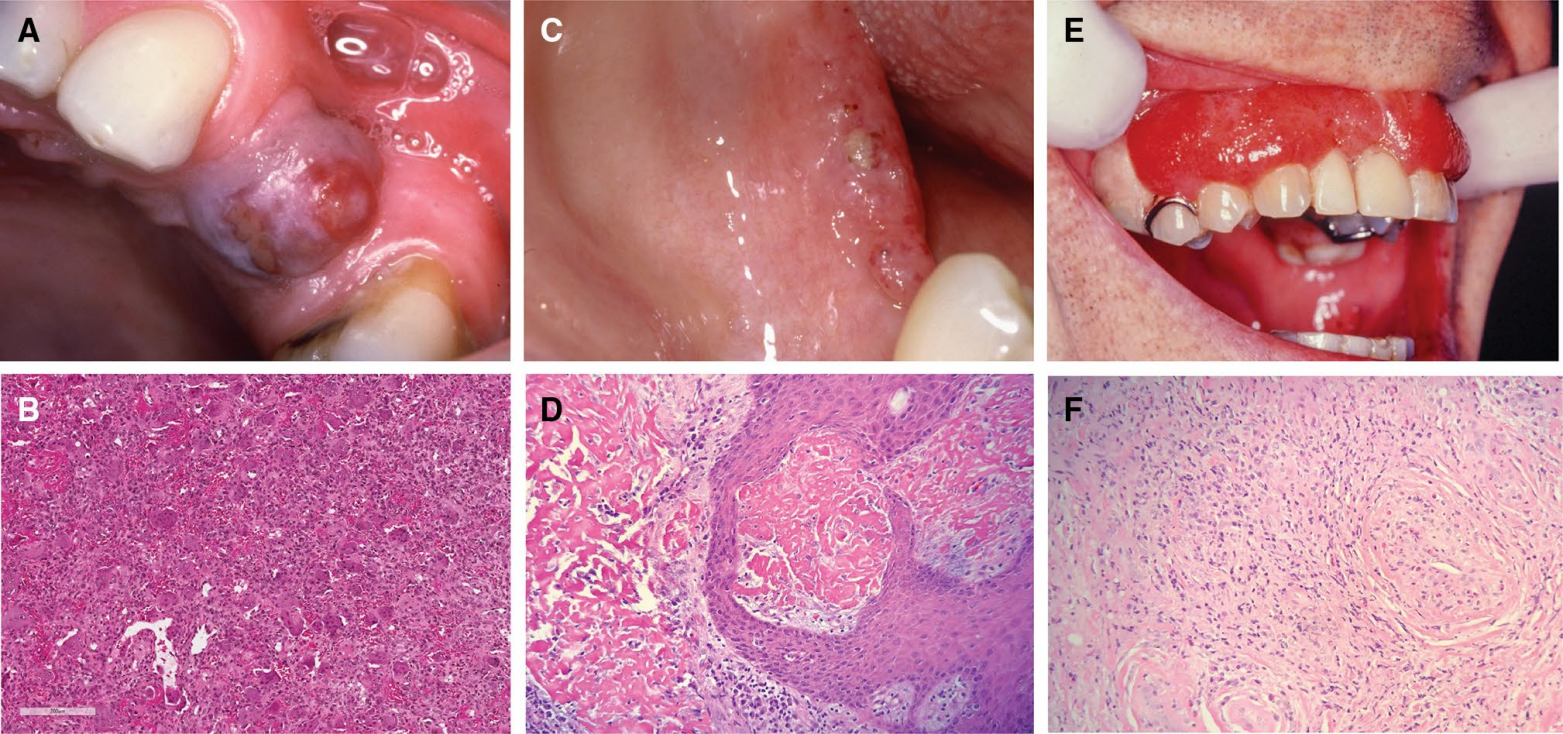
**a** A PGCG in an edentulous span of the maxilla. **b** Numerous multinucleated giant cells in a vascular and monocellular background in PGCG (H&E, Overall magnification × 40). **c** Lesions of ligneous alveolitis on the edentulous mandibular ridge. **d** Fibrinous deposits are seen in ligneous gingivitis, closely associated with the surface epithelium. Whilst suggestive of amyloid, these are Congo Red negative (H&E, overall magnification × 100). **e** Widespread “strawberry gingivitis” appearance of the maxillary gingiva in a patient with GPA. **f** The classic histological features of GPA can be difficult find in a gingival biopsy. The photomicrograph shows a small vessel with leukocytoclastic vasculitis and a poorly formed granuloma to the left of it (H&E, overall magnification × 100)

PGCG are most often deep red/purple colored sessile swellings that may reach an appreciable size (Fig. 2a). They may extend through the contact point between teeth in a dumb-bell type pattern. The clinical differential diagnosis includes ulcerated fibrous epulis and vascular epulis. The comments on generalized swelling in the fibrous hyperplasia section also apply.

#### Histology and Histological Differential Diagnosis

The main body of the lesion comprises a vascular and cellular stroma of mononuclear cells (fibroblasts, macrophages and endothelial cells), in which are scattered numerous multinucleated giant cells (Fig. 2b). The giant cells vary in size and number of nuclei. In some cases, fibrous septae are present and the lesional tissue is separated from the overlying epithelium by a band of fibrous tissue. Extravasated red blood cells and haemosiderin pigment are commonly identified.

PGCG are indistinguishable histologically from central giant cell granuloma and lesions of the jaws seen in hyperparathyroidism [13]. In many lesions, the initial excision is incomplete and the reporting pathologist should note this, together with a comment on the need to ensure that further investigations, such as radiological examination and, if deemed necessary, measurement of serum calcium levels are undertaken to exclude the aforementioned entities. The histological differential diagnosis of cherubism is rarely an issue due to the distinctive clinical and radiological features of that condition [14]. These include a family history, appearance of swellings early in life, and multiple radiolucent lesions of the jaws evident on radiological examination.

### Ligneous Gingivitis

#### Epidemiology

This rare lesion, caused by an inherited plasminogen deficiency, is seen in up to a third of such patients [15].

#### Clinical Presentation and Differential Diagnosis

The presentation is variable, from focal lesions to generalized nodular gingival enlargement. Patients may complain of bleeding and soreness. These lesions have an irregular surface and may ulcerate (Fig. 2c). An association with a form of aggressive periodontal disease has been reported.

#### Histology and Histological Differential Diagnosis

Fibrin accumulates in the superficial lamina propria, often associated with the vasculature in that area. If extensive, the fibrin can form large sheets, raising the suspicion of amyloid. The surface epithelium may be attenuated or mildly hyperplastic. There is often an associated scattered inflammatory infiltrate. The main histological differential diagnosis is that of amyloidosis. Histochemical stains to rule out amyloid (Congo or Sirius Red) and, on occasion, a trichrome stain (for example martius scarlet blue) may be useful in identification of the fibrinous material [16].

### Granulomatosis with Polyangiitis (GPA)

#### Epidemiology

GPA is a rare condition with a wide range of clinical presentations. Most are associated with the respiratory tract but any body system may be affected. The condition occurs over a wide age range with slight female predilection and almost all cases occur in Caucasians [17].

#### Clinical Presentation and Differential Diagnosis

Around 2% of patients present with oral lesions [18]. The classic oral presentation is that of ‘strawberry gingivitis’ (Fig. 2e). These lesions are nodular and erythematous with an irregular surface and a tendency to bleed easily. Lesions may be focal or widespread and may be asymptomatic. On occasion, there may be associated ulceration and destructive lesions have been reported [19]. The clinical appearance is characteristic, but the differential diagnosis may include some of the vascular lesions described above, particularly if the lesion is focal.

#### Histology and Histological Differential Diagnosis

The characteristic feature is of a destructive vasculitis (leukocytoclastic vasculitis: Fig. 2f). Affected vessels show inflammatory cells throughout the wall and these may be associated with vessel obliteration creating areas of necrosis. Gingival lesions also may show prominent red cell extravasation. Non-caseating epitheloid granulomas are also identified and these may contain multinucleated giant cells. Diagnosis can be challenging in small gingival biopsies due to the lack of granulomas and limited vasculitis. Other granulomatous conditions must be excluded, including tuberculosis, certain fungal infections, Crohn’s and sarcoidosis. Depending on the histological features, appropriate histochemical stains (Ziehl Neelsen, Grocott etc.) can be used to refine the differential diagnosis. Serological testing for PR3-ANCA should be suggested if GPA is in the differential diagnosis.

### Verruciform Xanthoma

This lesion is most common on the gingiva and the palate. As it often has a yellow coloration, the reader is referred to “Yellow lesions of the oral mucosa” and “Non-HPV Papillary lesions of the oral mucosa” in this special edition of the journal.

## Developmental Lesions

### Congenital Epulis

#### Epidemiology

The congenital epulis (syn: granular cell epulis) is a rare soft tissue lesion which most commonly develops on the anterior alveolar ridge of newborns. It is more common in females and in the maxilla.

#### Clinical Presentation

The lesion is usually present at birth and is a soft, mucosal covered nodule, varying in size from a few mm in diameter to several cm. Spontaneous resolution of lesions has been reported [20].

#### Histology and Histological Differential Diagnosis

The characteristic histological feature is a submucosal mass of large eosinophilic cells with granular cytoplasm. Pseudoepitheliomatous hyperplasia of the overlying epithelium is absent, a useful feature in distinction from granular cell tumor, which is the main differential diagnosis. These cells do not express S100 protein, again in distinction from cells in a granular cell tumor.

### Tori and Exostoses

#### Epidemiology

Torus palatinus (TP) and torus mandibularis (TM) are common findings within the oral cavity, with a prevalence of 12–15% [21]. They usually present in early adult life [22]. TP are twice as common in females, whereas TM show a slight male predominance [22]. Buccal and palatal exostoses are multiple bony nodules that occur less frequently than tori [23]. They are associated with increasing age and are more common in men than women [23].

#### Clinical Presentation and Differential Diagnosis

**Fig. 3:**
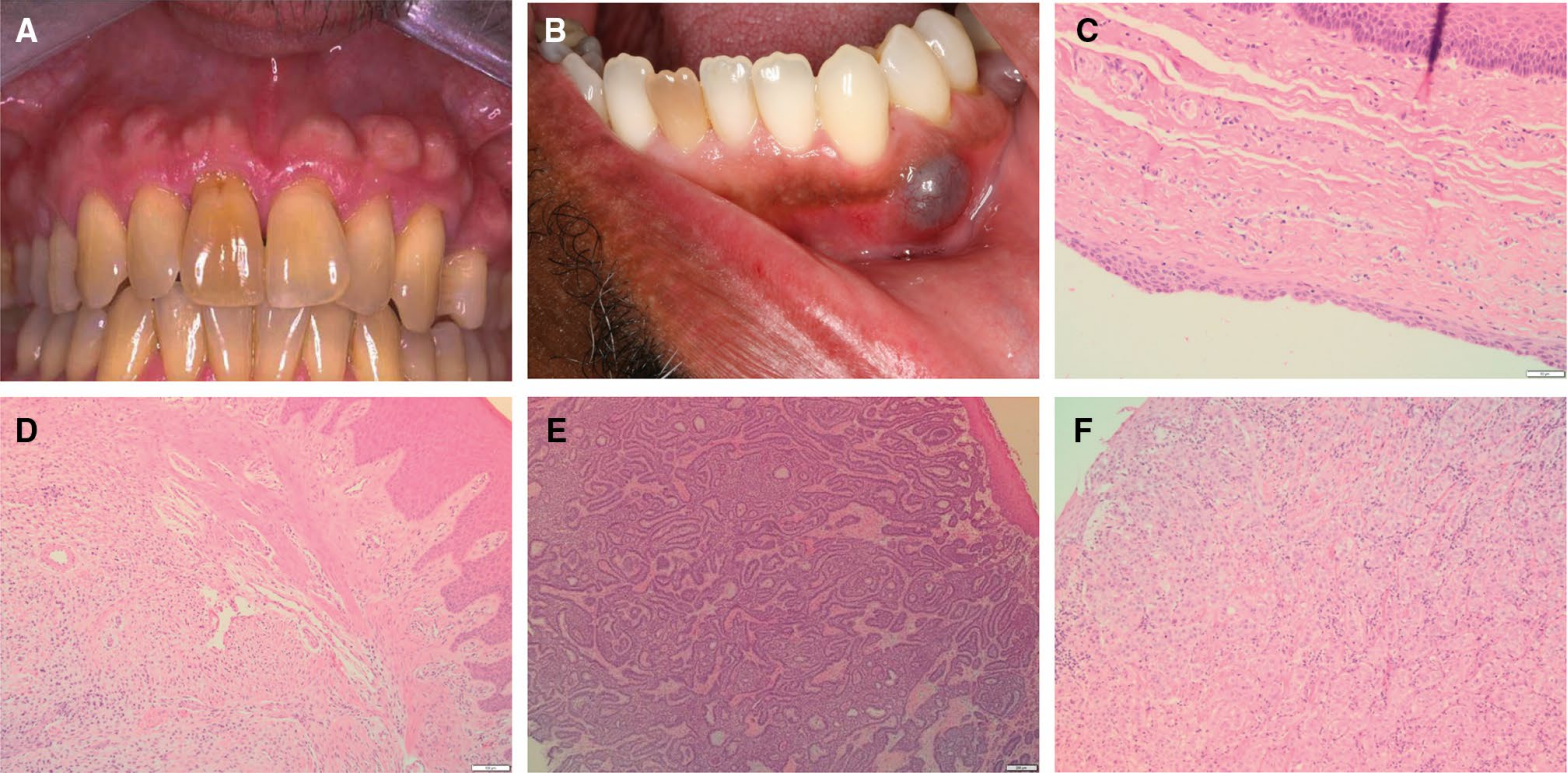
**a** Multiple bony swellings affecting the labial aspect of the maxillary gingivae, consistent with exostoses. **b** Bluish swelling affecting the attached gingivae in the lower left canine/premolar area, consistent with a gingival cyst (Photograph kindly provided by Dr Susan Muller). **c** Oral mucosa containing a cystic structure lined by thin epithelium with focal thickenings in a gingival cyst (H&E, overall magnification × 20). **d** An odontogenic fibroma is characterized by strands of odontogenic epithelium in a collagenous stroma (H&E, overall magnification × 10). **e** Peripheral ameloblastoma showing islands of odontogenic epithelium with characteristic peripheral palisading (H&E, overall magnification × 4). **f** Cords of atypical epithelial cells in fibrous stroma in a metastatic lobular carcinoma of breast (H&E, overall magnification × 20)

TP presents in the midline of the hard palate and TM presents on the lingual aspect of the mandible, above the mylohyoid line [21]. Exostoses are usually noted as multiple nodules on the buccal aspect of the maxilla (Fig. 3a) [23]. Their development is considered multifactorial, however TP and TM are associated with tooth attrition which has supported the theory that the mechanical stress of bruxism may play a part in the development of these lesions [21]. Tori and exostoses do not usually provide a clinical diagnostic dilemma.

#### Histology and Histological Differential Diagnosis

The histology for tori and exostoses is identical, so separation is made on clinical presentation [22]. Histology reveals mature lamellar bone, with scattered osteocytes and minimal osteoblastic activity [22]. Both cortical and trabecular bone are seen [22, 23].

### Gingival Cyst

#### Epidemiology

The gingival cyst of the adult is a relatively rare lesion, which accounts for a small percentage (0.2%) of odontogenic cysts [3]. It usually presents in the 6th decade of life and is more prevalent in women than in men [24].

#### Clinical Presentation and Differential Diagnosis

These lesions usually present as a painless, bluish swelling on the gingivae [25] and they are most frequently encountered in the incisor, canine and premolar regions of the mandible (Fig. 3b) [24]. The correct clinical diagnosis is made in about 50% of cases, however clinically a mucocele may be considered due to the bluish translucent appearance of a gingival cyst [26]. A radiograph would be required to confirm the soft tissue location and rule out an intraosseous process [24].

#### Histology and Histological Differential Diagnosis

These lesions are usually small and lined by a thin epithelium resembling reduced enamel epithelium, comprised of 1–3 layers of flat to cuboidal cells (Fig. 3c) [27]. The fibrous connective tissue wall is typically uninflamed and there is often separation between the epithelium and connective tissue [25]. Focal thickenings of the epithelium are frequently identified [25, 27].

Histologically, a gingival cyst is indistinguishable from a lateral periodontal cyst, therefore radiographic examination is required to exclude an intraosseous lesion [27]. Gingival cysts can cause resorption of the underlying cortical bone which may be seen as a diffuse radiolucency, however a lateral periodontal cyst will usually present as a well-defined radiolucency between the roots of the teeth [27]. Gingival cysts are usually unicystic but occasionally multicystic variants can be encountered [25] and if this is the case the botryoid odontogenic cyst, the multicystic variant of the lateral periodontal cyst, must be excluded.

## Neoplastic: Benign

### Peripheral Odontogenic Tumors

#### Epidemiology

Peripheral odontogenic tumors are rare lesions, with peripheral odontogenic fibroma (POF) and peripheral ameloblastoma (PA) occurring most commonly [28]. In one case series, peripheral odontogenic tumors accounted for 0.05% of oral biopsies and peripheral odontogenic tumors made up 4% of all odontogenic tumors [28]. POF is the most common odontogenic tumor to present peripherally and is in fact more common than its central counterpart [29]. It presents at a mean age of 32.3 years and has a slight female predominance [29]. PA presents at a mean age of 52.1 years, which is higher than its intraosseous counterpart [30]. It shows a slight male predominance [30].

#### Clinical Presentation and Differential Diagnosis

POF presents as a gingival swelling, usually with intact overlying mucosa [31]. It is more common in the mandibular incisor, canine and premolar area, whereas the central odontogenic fibroma is seen more often in the mandibular molar and premolar regions, as well as in the anterior maxilla [22, 29]. PA have a varying presentation and may have a granular or erythematous surface [30]. They are most common in the mandibular premolar region [30].

The clinical differential of a localized gingival mass is usually fibrous hyperplasia, a pyogenic granuloma or a giant cell lesion. If a PA has a very granular surface, then a squamous papilloma may also be considered in the clinical differential diagnosis [30]. An intraosseous odontogenic tumor presenting peripherally needs to be excluded with appropriate radiology [29], however peripheral lesions can cause superficial bone erosion [30].

#### Histology and Histological Differential Diagnosis

POF is microscopically similar to its central counterpart, characterised by a collagenous stroma containing bland appearing strands of odontogenic epithelium and possible hard tissue formation (Fig. 3d) [31]. PA can show any of the histopathological features that the intraosseous counterpart shows [31]. Typically, the follicular form is identified, comprising islands of odontogenic epithelium in a fibrous stroma [31]. The odontogenic epithelium resembles the enamel organ with peripheral palisaded ameloblast-like cells and a central stellate reticulum-like region (Fig. 3e) [31].

Sclerosing odontogenic carcinoma is a possible histological differential diagnosis for POF, however this would be more infiltrative and typically shows perineural invasion [31]. The differential diagnosis for a PA includes salivary gland tumors with similar histology and basal cell carcinomas [31], however oral mucosal involvement of basal cell carcinomas is rare. Immunohistochemistry is useful in cases where there is uncertainty.

## Neoplastic: Malignant

### Verrucous Carcinoma (VC)

#### Epidemiology

Within the oral cavity, the gingiva (12%) and buccal mucosa (10%) are the most common sites for VC [32] unlike conventional squamous cell carcinoma which is often found on the lateral border of the tongue and floor of mouth.

#### Clinical Presentation and Differential Diagnosis

VC presents as a thickened, white lesion with a roughened or papillary surface. Often, the degree of whiteness and hyperplasia varies within the same lesion. VC tends to grow slowly and does not metastasize. Clinically, VC is indistinguishable from verrucous hyperplasia [33] and often a spectrum of disease is present within the same lesion. Papillary squamous cell carcinoma may look similar. More subtle, small lesions may be confused with verruciform xanthoma, traumatic keratosis or papilloma.

#### Histology and Histological Differential Diagnosis

VC is characterized by extensive hyperplasia of rete processes that push deeply into the connective tissue, creating a buttress between the carcinoma and adjacent epithelium. There are varying degrees of hyperkeratosis and keratin plugging giving a verrucous surface morphology. Cellular and nuclear pleomorphism are minimal.

The classic histological conundrum is distinguishing VC from verrucous hyperplasia, especially on small or superficial biopsies. VC requires rete pegs to extend beneath the level of the adjacent epithelium, and without this buttress, it can be very difficult to make the distinction. A conventional squamous cell carcinoma may also have exo-endophytic qualities but contains significantly more cellular atypia and pleomorphism with foci of conventional tumor islands invading the superficial connective tissue.

### Metastases

#### Epidemiology

Metastatic disease to the oral cavity is often secondary spread from other metastatic lesions, most commonly the lungs [34, 35]. Metastases to the gingiva represent one of the most common sites for metastases to the oral cavity [34, 36–38]. Those aged between the 5th and 7th decades are most commonly affected with a male predominance (2:1 ratio) [39]. Metastases to the gingivae are most commonly from the lung, kidney and skin in men and the breast, genital organs and lung in women [39]. Metastases from the gastrointestinal tract and prostate are also possible.

#### Clinical Presentation and Differential Diagnosis

The clinical presentation is usually non-specific but the presence of swelling, ulceration and/or adjacent tooth mobility is common (Fig. 3a). Other malignant tumors, such as squamous cell carcinoma, lymphoma and melanoma, are often considered. When the patient has a history of a previous malignancy elsewhere in the body, the possibility of a metastasis should always be considered.

#### Histology and Histological Differential Diagnosis

It is not possible to discuss every type of tumor that may metastasize to the gingivae but the key is not to discount the possibility of metastases, especially when the morphology is unusual for those tumors that are more often seen in the head and neck region. Immunohistochemistry is a very useful adjunct for confirming the site of origin. The appearance of metastases varies greatly depending on the site of origin of the metastatic tumor. For instance, a renal cell carcinoma metastasis consists of sheets of bland clear cells in a vascular stroma (typically RCC, CD10, PAX8 positive). In the gingiva, clear cell carcinoma and clear cell odontogenic carcinoma would need excluding (CD10, PAX8, RCC negative). Lobular carcinoma of the breast tends to have cords of cells similar to polymorphous adenocarcinoma, however the former is usually ER and PR positive unlike the latter (Fig. 3f).

### Kaposi’s Sarcoma (KS)

#### Epidemiology

KS is always associated with HHV8 infection and has four epidemiological categories, but the AIDS-related type is the only one associated with oral manifestations [40]. Oral KS is most common in the 4th and 5th decades affecting the palate and gingiva the most commonly.

#### Clinical Presentation and Differential Diagnosis

**Fig. 4:**
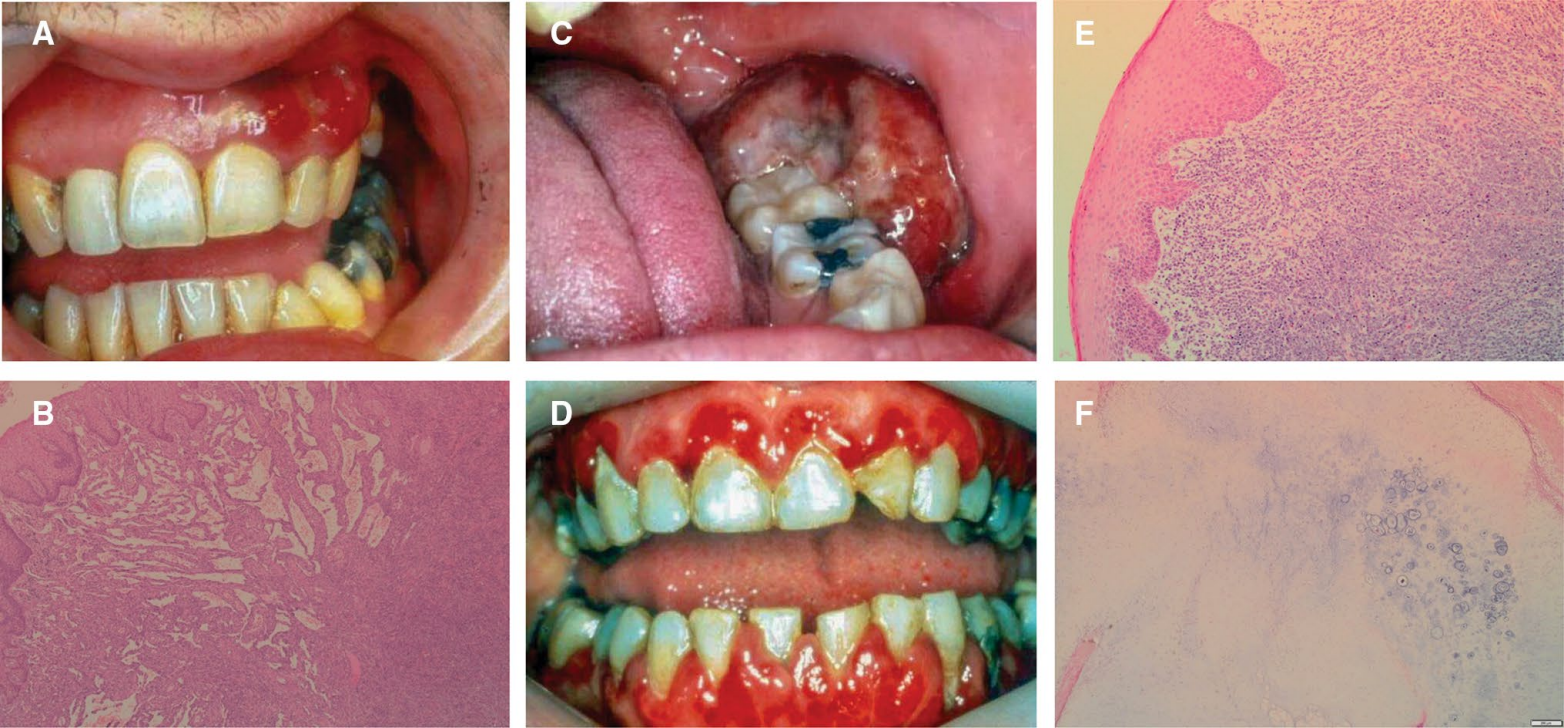
**a** Red, nodular swelling affecting the facial gingiva above the left maxillary canine and lateral incisor in Kaposi Sarcoma. **b** Streams of spindled cells with slit-like vessels and lymphangiomatous pattern superficially in Kaposi’s sarcoma (H&E, overall magnification × 4). **c** Non-Hodgkin lymphoma presenting as an ulcerated swelling affecting the posterior left retromolar region. **d** Generalized erythema and swelling affecting the gingiva in a case of AML. **e** Connective tissue effaced by sheets of atypical myeloid cells in AML (H&E, overall magnification × 20). **f** Chondrosarcoma classically has a lobular architecture, with blue-grey cartilaginous matrix (H&E, overall magnification × 4)

Lesions vary from being subtle areas of discolouration (often red/purple macules and papules) to more extensive nodular lesions that have a more sinister appearance (Fig. 4a). The differential diagnosis often includes vascular lesions, such as haemangioma, pyogenic granuloma and giant cell epulis, especially if the lesion is nodular in appearance. Advanced lesions become large and ulcerated where other malignant differentials may be considered such as squamous cell carcinoma.

#### Histology and Histological Differential Diagnosis

The early lesion (patch stage) is fairly subtle and composed of thin, slit-like vessels within the lamina propria that transect collagen fibers and are seen in association with extravasated red blood cells and lymphocytes. In the plaque stage, the spindle-cell proliferation is more obvious and associated with hyaline globules (Kamino bodies). The nodular stage comprises sheets of spindle cells, which appear atypical with increased mitoses (Fig. 4b). The tumor cells are positive for endothelial markers such as CD31 and CD34, but are characteristically positive for HHV8.

The early features of KS can be easily missed. For instance, the increase in vessels may be mistaken for granulation tissue in relation to recent ulceration or trauma. When the tumor enters the more cellular phases, the histological differential diagnoses to consider are other spindle cell neoplasms with vasoformative qualities such as angiosarcoma (HHV8 negative).

### Lymphoma/Leukemia

#### Epidemiology

The head and neck is the second most common extra-nodal site for lymphoma (11–33%), typically affecting patients over 50 years old [41]. Intraorally, the most common sites affected are the vestibule, gingiva, mandible, palate, maxilla and tongue [42–46]. The majority are Non-Hodgkin B cell lymphomas (mostly diffuse large B-cell lymphoma) but T-cell lymphomas account for 12% of oral lymphomas in the Japanese population [44, 47]. The most common type of leukemia to affect the gingivae is acute myeloid leukemia (AML) of monocytic derivation [48].

#### Clinical Presentation and Differential Diagnosis

Lymphoma and leukemia have a non-specific clinical presentation, but often present with swelling and reddening of the gingival tissues (Fig. 4c, d). Advanced cases are likely to be accompanied by bone loss and tooth mobility. Unless the patient has a known history of lymphoma or leukemia, the differential diagnosis is likely to include a range of non-neoplastic and neoplastic conditions depending on the extent of disease at presentation [49]. When lesions are diffuse and present with reddening and swelling of the gingivae, conditions such as GPA, periodontitis and hyperplastic gingivitis may be considered. More localized swelling may be mistaken for pyogenic granuloma or giant cell epulis.

#### Histology and Histological Differential Diagnosis

The range of morphological features present in lymphomas and leukemia is beyond the scope of this article, but the key feature is that the normal connective tissue is effaced by atypical lymphoid/myeloid cells (Fig. 4e). The atypical cells are often arranged in sheets with high-grade lesions exhibiting obvious mitoses, nuclear and cellular pleomorphism and necrosis. Indolent B-cell lymphomas may be more monotonous in appearance but are less common in the gingiva. A basic immunohistochemical panel of CD20 and CD3 will highlight the lack of a mixed population in lymphomas. Referral to a haematopathologist is necessary for definitive subtyping.

If the lymphoma/leukemia is high grade, a reactive process is unlikely to be considered. Sometimes, there can be surface ulceration or co-existing periodontal disease which obscures the neoplastic infiltrate meaning careful attention must be paid to the clinical presentation and cytology.

### Osteosarcoma and Chondrosarcoma

#### Epidemiology

Osteosarcoma accounts for approximately 1% of all head and neck cancers [50] and the jaw bone are the 4th most common site for osteosarcoma [31]. Head and neck osteosarcoma occurs at a later age than it’s peripheral counterparts, with a median age of 36 years [31, 50]. Chondrosarcomas are rare, accounting for 0.1% of all head and neck neoplasms [51] and 3–4% of all chondrosarcomas [31]. They usually occur in middle age and are more common in males than in females [22, 31].

#### Clinical Presentation and Differential Diagnosis

The presentation of head and neck osteosarcoma depends on the location of the tumor, with most patients presenting with a mass alongside pain, possible paresthesia and loosening of teeth [31, 50]. Radiographically, an ill-defined mixed radiolucent and radiopaque lesion is seen, occasionally with the classical “sunburst” appearance [52]. Chondrosarcoma shows a similar clinical picture, with swelling being the primary presentation and other symptoms are specific to the location of the tumor, such as cranial nerve dysfunction, loose teeth and pain [31, 51].

Based on the radiology, the clinical differential for an osteosarcoma may include osteomyelitis [52], and in some cases without significant bony destruction, the possibility of a benign cemento-osseous lesion may be raised [53]. The clinical differential for a chondrosarcoma may include osteosarcoma or another more common malignant tumor such as a squamous cell carcinoma [22].

#### Histology and Histological Differential Diagnosis

Essential to the diagnosis of osteosarcoma is the presence of neoplastic bone, which is characteristically lace-like, woven in nature and closely associated with the tumor cells [54]. The tumor cells show significant pleomorphism and may be epithelioid, plasmacytoid, spindled or fusiform [54]. Histological subtypes of osteosarcoma include chondroblastic, fibroblastic and osteoblastic [54].

Chondrosarcoma of the head and neck shows similar histology to those found elsewhere in the body, with lobules of blue-grey cartilaginous matrix which may be separated by fibrous bands and can show calcification [54] (Fig. 4f). They are graded from I to III based on their level of cellularity, mitoses and cellular atypia [54].

The main histological differential diagnoses for osteosarcomas include osteoblastoma and chondrosarcoma [22]. Correlation with the radiology is useful in the case of an osteoblastoma, which should be circumscribed with a sclerotic margin [22]. It is also important to differentiate between chondrosarcoma and chondroblastic osteosarcoma, due to the improved prognosis of a chondrosarcoma [55] and because of the differing treatments of these two entities. Chondroblastic osteosarcomas can have an abundant chondroid component, however production of malignant osteoid by mesenchymal cells is diagnostic of osteosarcoma [55].

## Conclusion

The clinical and histological features of lumps and bumps are remarkably varied, encompassing much of any standard textbook “surgical sieve”. We have sought to outline these lesions, highlighting areas of difficulty and diagnostic pitfalls. As most of these lesions are reactive, close communication with the referring clinicians is required to ensure that appropriate management plans are enacted, including removal of the initiating factors. Nevertheless, it is important to keep rarer diagnoses in mind, as some represent disease processes with significance well beyond the gingiva.
